# Grading Mitral Regurgitation: To Severe and Beyond!

**DOI:** 10.1016/j.shj.2025.100425

**Published:** 2025-02-17

**Authors:** Amar Krishnaswamy, Rhonda Miyasaka

**Affiliations:** a Interventional Cardiology, Sones Cardiac Catheterization Laboratories, Cleveland Clinic, Cleveland, Ohio, USA; b Cardiovascular Imaging, Cleveland Clinic, Cleveland, Ohio, USA

The grading of valvular regurgitation is a crucial, yet sometimes challenging, step in the management of patients with mitral regurgitation (MR). In addition to understanding the pathologic basis of regurgitation, accurate quantification of MR drives decision-making regarding the need for intervention, defines procedural success, and predicts clinical benefit.

Currently, MR is classified as mild (1+), moderate (2+), moderate-severe (3+), or severe (4+).^1^ While advances in echocardiographic technology have led to additional metrics and methods to aid in MR quantification, the basic grading scale has largely remained unchanged and relies on both qualitative and quantitative assessments. Patients with severe MR are considered for intervention, and, while key trials for mitral transcatheter edge-to-edge repair (M-TEER)^2^^,^^3^ have targeted a goal result of MR 2+ or less, there is growing literature that even further MR reduction is associated with better clinical outcomes.^4^

In recent years, an expanded grading scale for the assessment of tricuspid regurgitation (TR) was proposed, adding the categories of “massive” and “torrential” beyond severe.^5^ This was based on the recognition that patients enrolling in early tricuspid clinical trials had baseline TR that was well above the cutoff for severe, and although TR reduction was achieved, it was difficult to qualify the degree of improvement, as both preprocedure and postprocedure TR fell in the “severe” range. For example, a patient with a baseline effective regurgitant orifice area of 90 mm^2^ (cutoff of severe >40 mm^2^), could have significant TR reduction to an effective regurgitant orifice area (EROA) of 45 mm^2^ postprocedure, but with the classic grading scale, both pre- and post-TR would fall in the severe range. While not yet recognized in society guidelines, the new scheme has been used in key contemporary transcatheter tricuspid valve trials.^6^^,^^7^ In the Triluminate study, this expanded grading scale allowed for granular assessment of quality of life outcomes related to TR reduction, finding that every 1-grade improvement in TR correlated with a 4.1-point increase in Kansas City Cardiomyopathy Questionnaire (KCCQ) score, even when pre- and post- would have both landed in the severe range of the historic scale.^8^

In the February issue of Structural Heart, a study by Narang et al. raised the question of whether MR could similarly benefit from an expanded grading scale and whether procedural outcomes were affected by the same. In this study, 225 patients who underwent M-TEER between July 2014 and March 2021 were retrospectively identified with the aim of reclassifying severe MR according to a proposed expanded grading scale. MR was graded via 2D EROA and regurgitant volume based on the proximal isovelocity surface area (PISA) method. The final cohort included 142 patients, after 48 were excluded for baseline MR less than severe (moderate-severe), and 35 were excluded for lack of quantifiable MR on review of available images. The etiology of MR was primary in 77% of patients, 14% secondary, and 9% mixed.

Using the expanded grading scale, 59% of patients remained in the severe range, while 23% were regraded as massive and 18% as torrential. In addition to MR reduction, baseline and post-transcatheter edge-to-edge repair left ventricular ejection fraction, left ventricular volumes, and health status (KCCQ-overall score and New York Heart Association class) were evaluated. As expected, KCCQ-overall score and New York Heart Association of the entire cohort improved postprocedure; however, there was no statistical significance between subgroups.

There are several significant limitations to this study that must be recognized. First, the sample size is very small, with only 142 patients over a 7-year time frame; numerous device and imaging iterations over this time have affected both assessment and procedural performance and precluded a homogenous grouping together. Primary and secondary MR were evaluated as a single cohort, though these 2 disease processes vary significantly both anatomically and clinically. Furthermore, MR grading was performed using a single variable, namely a PISA-derived EROA and regurgitation fraction, which has many known limitations. In primary MR, PISA may lead to overestimation of MR in the presence of nonholosystolic MR, while conversely, in secondary MR, PISA underestimates MR severity due to a broad jet of MR across the coaptation zone. For these reasons, current American Society of Echocardiography guidelines recommend grading MR severity based on multiple qualitative and quantitative metrics. More recently, 3-dimensional techniques have also been used for the pre- and post-assessment of MR, namely the measurement of 3-dimensional vena contracta area.^9^ Finally, the lack of any clinical difference in outcome may also be the result of the limited number of patients or selection bias surrounding the same, the previously stated limitations in the quantification of the MR degree, or other unmeasured factors.

On the other hand, while the echocardiographic and clinical significance of the current study is limited, the question of whether the MR grading scale should be expanded does have merit. Similar to what is seen in patients with TR, there is likely also a range of MR that is more than “severe” based on traditional metrics. For primary MR, this could include a patient with multiple degenerative segments, with each individually measured jet of MR falling in the severe range, compared to a patient with a focal jet of severe MR (Figure 1). For secondary MR, this might be a patient with leaflet noncoaptation and wide-open MR across the coaptation zone as opposed to a patient with a small coaptation gap and MR primarily along A2-P2 (Figure 2). The current grading scale does not allow for differentiating between these 2 types of “severe” MR, but clinically the differences could be relevant.

**Figure 1:**
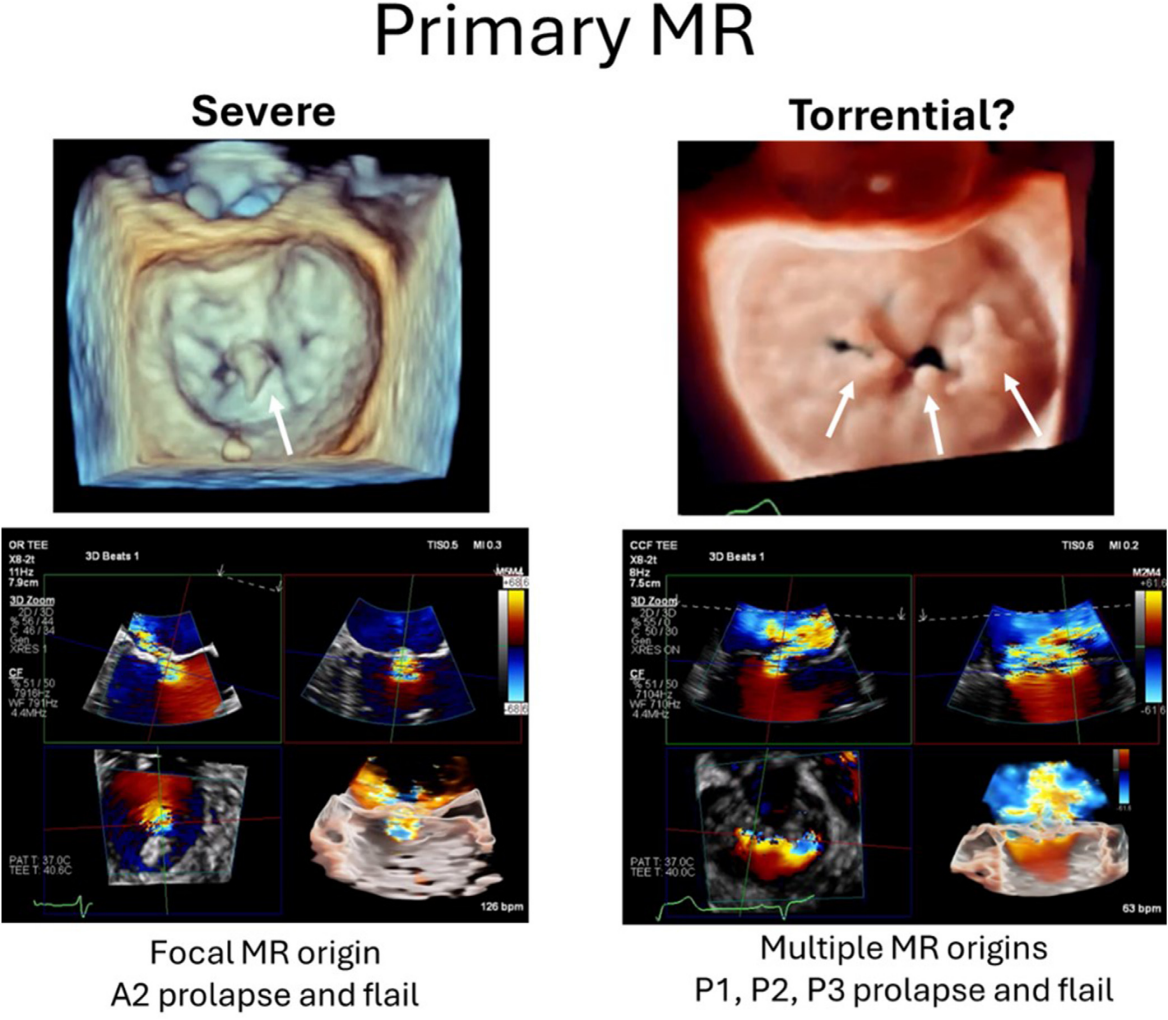
Severe vs. “beyond severe” primary mitral regurgitation.

**Figure 2:**
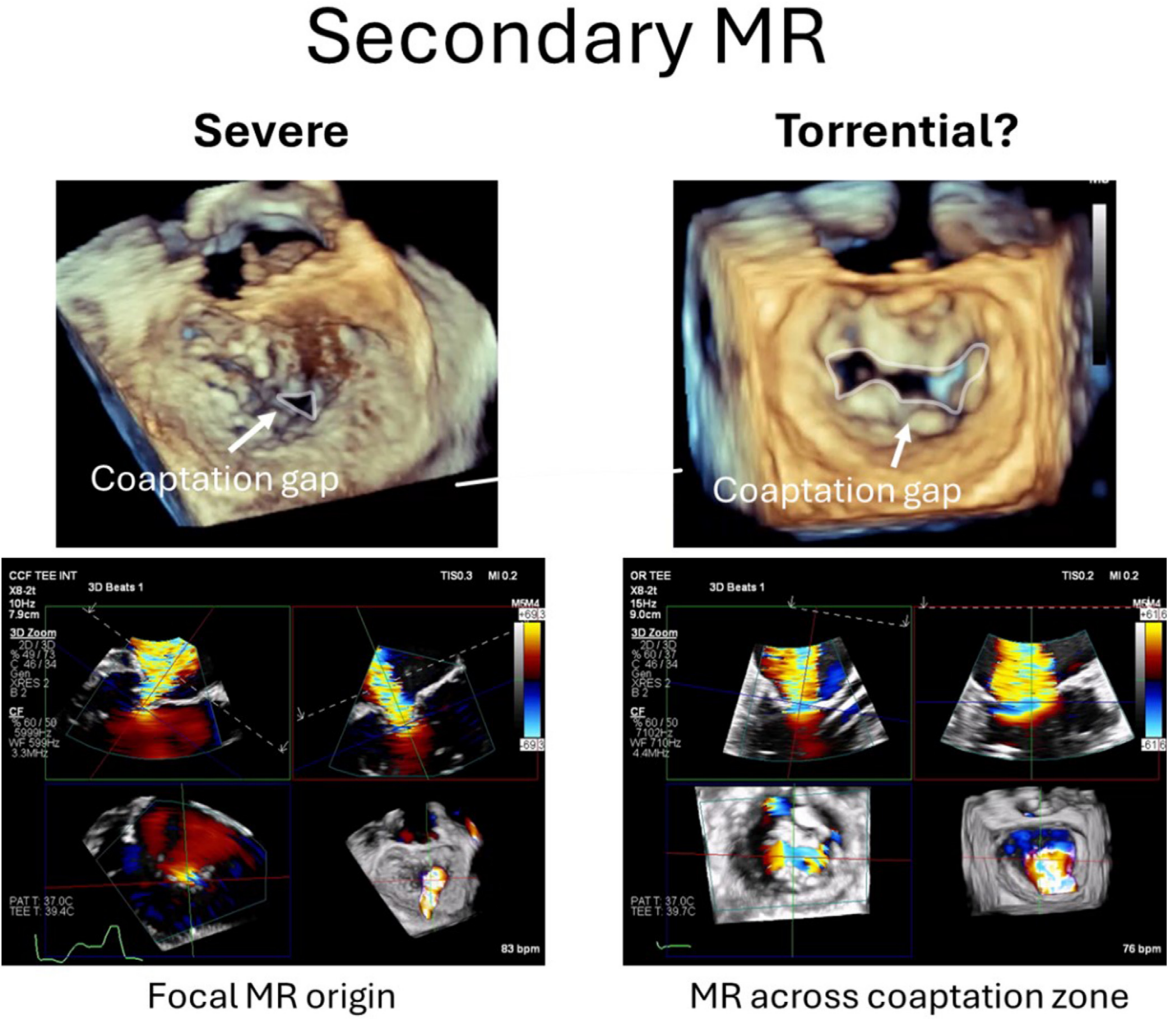
Severe vs. “beyond severe” secondary mitral regurgitation.

One such scenario would be the decision of whether to offer a mitral intervention when the expected result is suboptimal (i.e., more than mild residual MR). For example, the anatomy shown in Figure 1 is not ideal for transcatheter edge-to-edge repair; there are multiple diseased segments, only a portion of which can be treated before the development of mitral stenosis. Ideally, the patient would undergo evaluation for open heart surgery or a transcatheter mitral valve replacement; however, if these options are not available, should M-TEER be offered, even if the patient is expected to be left with moderate or severe MR by echocardiographic parameters? Should the patient expect clinical benefit? We have seen in the literature surrounding tricuspid valve-TEER that in addition to the final absolute TR grade, the degree of TR reduction also correlates to clinical benefit, and this distinction is made possible with the more granular TR grading scale. It would therefore be challenging to study these questions without recognition of MR grades above severe.

With respect to secondary MR, expanding the grading scale could help us differentiate between symptoms related to left ventricle dysfunction vs. symptoms related to severe MR, better understanding the concept of “proportionate” vs. “disproportionate” MR.^10^ When comparing the results of the MITRA-FR Trial^11^ vs. the Cardiovascular Outcomes Assessment of the MitraClip Percutaneous Therapy for Heart Failure Patients with Functional Mitral Regurgitation Trial,^12^^,^^13^ one of the key differences was that the degree of MR was more severe in Cardiovascular Outcomes Assessment of the MitraClip Percutaneous Therapy for Heart Failure Patients with Functional Mitral Regurgitation Trial. Is it possible that if we further stratified severe MR, we could better understand which patients will achieve the most benefit from M-TEER?

While expanding the grading scale seems to offer many potential benefits, we must also recognize the associated challenges, the most important of which is that we still do not have a single, reliable, reproducible metric for quantitatively determining MR severity. All of the current metrics have limitations, which is why guidelines recommend taking into consideration multiple quantitative and qualitative parameters. It is hard to understand how to expand the current grading scale when there is difficulty using the current, simpler one.

As a provocative idea, we could consider including invasive hemodynamics as part of the MR severity assessment, such as baseline left atrium pressure and V wave. Clinically, hemodynamic data are often used intraprocedurally to understand the degree of MR reduction and potential clinical benefit, especially among those patients where real-time assessment to balance MR reduction with creation of mitral stenosis is necessary. It seems reasonable that such metrics could be included in the baseline assessment of MR severity. Perhaps it is time to rethink MR grading, not just in the scale that is used, but the metrics that are incorporated: blending imaging and invasive hemodynamic parameters, mirroring the teamwork between imaging and intervention during these procedures.

While an expanded grading scale may assist in future research, we must also be cautious that this does not undermine the importance of achieving the greatest degree of MR reduction. In both surgical and percutaneous literature, it has been shown that residual MR is associated with worse outcomes.^14^ A challenge with M-TEER is the intraprocedural decision-making regarding placing additional devices for further MR reduction, balanced against the risk of mitral stenosis. An expanded grading scale could make it tempting to leave behind significant residual MR if the starting grade is torrential. This may be acceptable in limited clinical scenarios, but this is a slippery slope when evaluating patients who potentially have several different percutaneous or surgical options. For this reason, if an expanded grading scale were pursued, it would be important that this be used as a research tool only until further data become available to understand clinical relevance.

By raising the question of an expanded MR grading scale, the study by Narang et al*.* highlights key issues regarding patient selection and predictors of success for patients undergoing M-TEER. More research is necessary to determine the optimal methods for quantifying MR severity and its relationship to clinical outcomes, but it seems reasonable that an expanded grading scale could be beneficial to the field.

## Funding

The authors have no funding to report.

## Disclosure Statement

The authors report no conflict of interest.
